# *CDKN2B* methylation is associated with carotid artery calcification in ischemic stroke patients

**DOI:** 10.1186/s12967-016-1093-4

**Published:** 2016-12-01

**Authors:** Shuyu Zhou, Yumeng Zhang, Li Wang, Zhizhong Zhang, Biyang Cai, Keting Liu, Hao Zhang, Minhui Dai, Lingli Sun, Xiaomeng Xu, Huan Cai, Xinfeng Liu, Guangming Lu, Gelin Xu

**Affiliations:** 1 Department of Neurology, Jinling Hospital, Medical School of Nanjing University, Nanjing, 21002 China; 2 Department of Gerontology, Nanjing Drum Tower Hospital, Medical School of Nanjing University, Nanjing, 21008 China; 3 Department of Medical Imaging, Jinling Hospital, Medical School of Nanjing University, Nanjing, 21002 China; 4 Department of Neurology, Jinling Hospital, Southern Medical University, Nanjing, 21002 China

**Keywords:** Carotid artery calcification, *CDKN2A/2B*, DNA methylation, Ischemic stroke

## Abstract

**Background:**

Cyclin-dependent kinase inhibitor 2A/2B (*CDKN2A/2B*) near chromosome 9p21 have been associated with both atherosclerosis and artery calcification, but the underlying mechanisms remained largely unknown. Considering that *CDKN2A/2B* is a frequently reported site for DNA methylation, this study aimed to evaluate whether carotid artery calcification (CarAC) is related to methylation levels of *CDKN2A/2B* in patients with ischemic stroke.

**Methods:**

DNA methylation levels of *CDKN2A/2B* were measured in 322 ischemic stroke patients using peripheral blood leukocytes. Methylation levels of 36 CpG sites around promoter regions of *CDKN2A/2B* were examined with BiSulfite Amplicon Sequencing. CarAC was quantified with Agatston score based on results of computed tomography angiography. Generalized liner model was performed to explore the association between methylation levels and CarAC.

**Results:**

Of the 322 analyzed patients, 187 (58.1%) were classified as with and 135 (41.9%) without evident CarAC. The average methylation levels of *CDKN2B* were higher in patents with CarAC than those without (5.7 vs 5.4, *p* = 0.001). After adjustment for potential confounders, methylation levels of *CDKN2B* were positively correlated with cube root transformed calcification scores (β = 0.591 ± 0.172, *p* = 0.001) in generalized liner model. A positive correlation was also detected between average methylation levels of *CDKN2B* and cube root transformed calcium volumes (β = 0.533 ± 0.160, *p* = 0.001).

**Conclusions:**

DNA methylation of *CDKN2B* may play a potential role in artery calcification.

**Electronic supplementary material:**

The online version of this article (doi:10.1186/s12967-016-1093-4) contains supplementary material, which is available to authorized users.

## Background

As a surrogate measure of atherosclerosis, calcification may contribute to plaque vulnerability and, therefore, risk of vascular events [1]. Because carotid bifurcation and adjacent segments are the predilection sites of atherosclerosis, calcification in these location can reflect the overall burden of vascular calcification [2], and may predict risk of stroke, myocardial infarction and the overall vascular events [3, 4].

**Fig. 1:**
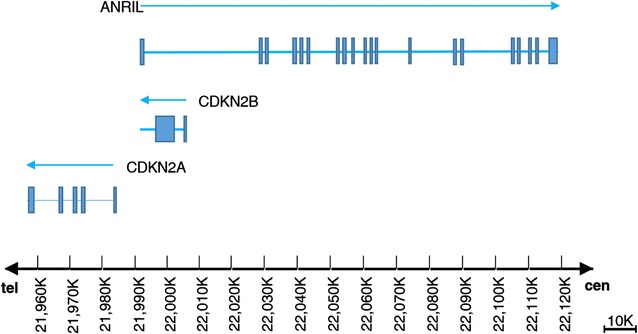
Illustration of genomic organization of the 9p21 locus. *Blue lines* with *arrows* represent the approximate locations and transcribe directions of *CDKN2A*, *CDKN2B* and *ANRIL*. *Blue boxes* indicate *exons*. *ANRIL* is transcribed in opposite direction of *CDKN2A/2B* genes. Cen indicates centromere, and tel indicates telomere

Genetic factors have long been proposed with an important role in the initiation and development of arterial calcification [5, 6]. After the landmark genome-wide association studies identified human chromosome 9p21 (Chr9p21) as a potential genetic origin both for atherosclerosis and artery calcification [7–9], determining gene variants responsible for artery calcification has become a focus of many studies. Intriguingly, Chr9p21 region is actually a “gene desert” devoid of annotated protein-coding genes. Only the antisense noncoding RNA in the INK4 locus (*ANRIL*) is transcribed in this region. The closest protein-coding genes to Chr9p21 locus are two cyclin-dependent kinase inhibitors, *CDKN2A* and *CDKN2B*, both of which involve in cell cycle regulation (Fig. 1). This locational neighborhood between Chr9p21 and *CDKN2A/2B* may suggest their functional associations, which have been evidenced by results from recent studies [10, 11]. For example, Motterle et al. showed that Chr9p21 variation can change the level of ANRIL transcription, which in turn alter expression of *CDKN2A/2B* and enhance proliferation of vascular smooth muscle cells (VSMCs), and subsequently promote atherosclerosis [11].

Both functional [12] and genetic studies [13, 14] suggested that *CDKN2A/2B* may promote atherosclerosis by facilitating the process of calcification. But the mechanisms remain largely unknown. Considering that *CDKN2A/2B* is a frequently reported site of action for DNA methylation [15, 16], we hypothesized that DNA methylation in *CDKN2A/2B* may increase the susceptibility of artery calcification. In this study, we tested this hypothesis by evaluating the degree of DNA methylation in *CDKN2A/2B* and the carotid calcification load in a cohort of patients with ischemic stroke.

## Methods

### Study population

This study was approved by the Ethical Review Board of Jinling Hospital. Written informed consent was obtained from all enrolled patients. Consecutive patients with ischemic stroke were screened from Nanjing Stroke Registry Program [17] between July 2012 and September 2013. Patients were included if they: (1) were diagnosed with first-ever ischemic stroke within 7 days of onset; (2) aged 18 years or older; (3) completed a neck computed tomography angiography (CTA). Ischemic stroke was diagnosed if there were new focal neurological deficits explained by relevant lesions detected on diffusion-weighted imaging or computed tomography. Patients with malignant neoplasm, severe liver or kidney dysfunction, autoimmune diseases, parathyroid gland diseases, or calcium-phosphorus metabolic disorders were excluded. Since the stents may influence the accuracy of calcification assessment, patients with history of carotid artery stenting were also excluded. A total of 391 patients were screened and 324 patients were finally enrolled.

### Artery calcification measurement

Each enrolled patient underwent a neck computed tomography angiography for CarAC evaluation. CTA was performed by a dual-source 64 slice CT system (Siemens, Forchheim, Germany) to quantify CarAC. Imaging was acquired by scanning from 4 cm below aortic arch to the superior border of orbit in craniocaudal direction. Details on CTA scan have been provided elsewhere [18].

Calcification scores in carotid artery were measured with Syngo Calcium Scoring system (Siemens, Forchheim, Germany). A focus of ≥4 contiguous pixels accompanied by a CT density ≥130 Hounsfield units (HU) was defined as calcification according to the method of Agatston score [19]. Area of calcification (mm^2^) was multiplied by a weighted value assigned to its highest HU (130–199HU = 1; 200–299HU = 2; 300–399HU = 3; and >400HU = 4). Carotid calcification was measured at both sides within 3 cm proximal and distal segments of the bifurcation including four artery segments: common, bulb, internal, and external. The software used for calculating Agatston score also provided an isotropically interpolated calcium volume (mm^3^), by calculating the numbers of voxels with attenuation ≥130HU and summing the total voxel volumes. Calcification scores and calcium volume were assessed by two raters independently. The raters were blinded to other clinical data.

### DNA isolation and epi-genotyping

Venous blood samples were drawn in the morning after an overnight fasting for biochemical marker assaying and methylation analyzing. Genomic DNA was extracted from whole blood with commercially available kits (TIANGEN Biotech, Beijing, China). DNA was quantified and then diluted to a working concentration of 10 ng/μL for genotyping.

**Fig. 2:**
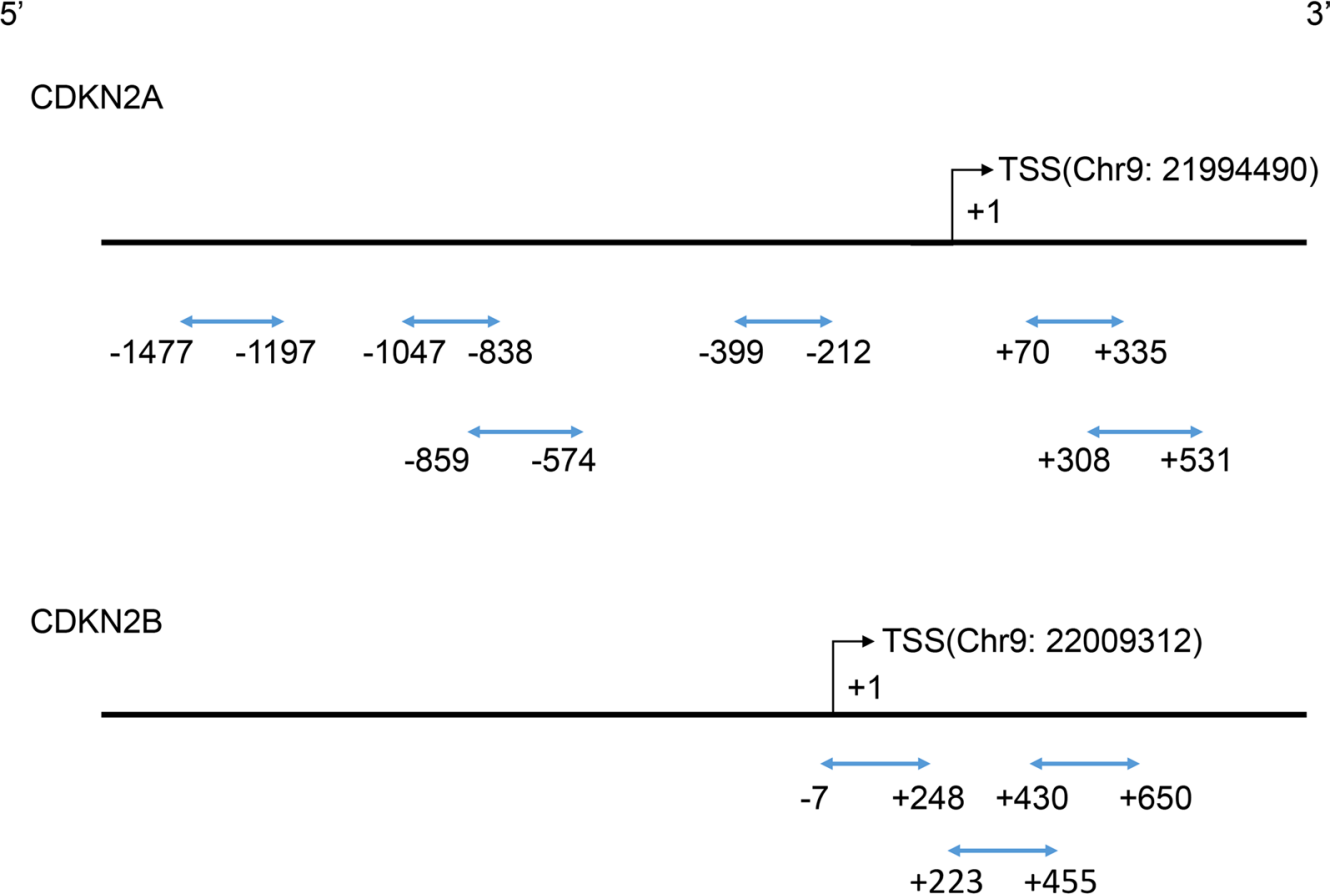
CpG regions sequenced around promoter of *CDKN2A/2B. Blue lines* with *arrows* indicate selected CpG regions analyzed in this study, all of which locate in CpG islands around gene promoters. Range of each region is indicated by its relative distance (in bp) to TSS

CpG islands located in the proximal promoter of *CDKN2A/2B* were selected for measurement according to the following criteria: (1) 200 bp minimum length; (2) 50% or higher GC content; (3) 0.60 or higher ratio of observed/expected dinucleotides CpG. Six regions from CpG islands of *CDKN2A* and three from that of *CDKN2B* were selected and sequenced (Fig. 2). BiSulfite Amplicon Sequencing (BSAS) was used for quantitative methylation analysis [20]. Bisulfite conversion of 1 μg genomic DNA was performed with the EZ DNA Methylation™-GOLD Kit (ZYMO RESEARCH, CA, USA) according to the manufacturer’s protocol. Sodium bisulfite preferentially deaminates unmethylated cytosine residues to thymines, whereas methyl-cytosines remain unmodified. After PCR amplification (HotStarTaq polymerase kit, TAKARA, Tokyo, Japan) of target CpG regions and library construction, the products were sequenced on Illumina MiSeq Benchtop Sequencer (CA, USA). Primer sequences used for PCR were shown in Additional file 1: Table S1. All samples achieved a mean coverage of >600X. Each tested CpG site was named as its relative distance (in bp) to transcriptional start site (TSS). Methylation level at each CpG site was calculated as the percentage of the methylated cytosines over the total tested cytosines. The average methylation level was calculated using methylation levels of all measured CpG sites within the gene.

### Statistical analysis

Normality of parameters was assessed by Shapiro–Wilk test. As all continuous data in this study did not meet the normality assumption, they were described as median (interquartile range) and compared with Mann–Whitney U test. The non-parameters were compared with Fisher’s exact test. Patients were classified as without (Agatston score = 0), with mild (0 < Agatston score ≤ 100) and with severe (Agatston score > 100) CarAC. Methylation levels of *CDKN2A/2B* were compared between patients with and without CarAC using Mann–Whitney U test. Methylation levels of *CDKN2A/2B* were also compared among patients with mild, severe and without CarAC using Kruskal–Wallis test.

Spearman correlations were used to evaluate pairwise correlations of methylation levels between different CpG sites in the same gene. Given the heavily skewed distribution of calcification scores and calcium volume, cube root transformation was performed before comparison, as suggested in the previous studies [21, 22]. Generalized linear model was used to explore the association between methylation levels and cube root transformed calcification scores/calcium volumes after adjusting for age, sex, body mass index (BMI), diabetes mellitus (DM), hypertension (HTN) and smoking. These variables were chose for adjustment because they were identified as confounders that affected artery calcification. Bonferroni correction was used for multiple testing.

The data were analyzed by IBM SPSS Statistics Version 22.0 (Armonk, NY: IBM Corp.). A two-tailed value of *p* < 0.05 was considered statistically significant.

## Results

Of the 324 enrolled patients, 2 (0.6%) failed in epi-genotyping. Finally, 322 (99.4%) patients were included for data analysis. Demographic characteristics and major risk factors for cardiovascular diseases were listed in Table 1. The median age of the 322 analyzed patients was 62.0 (55.0–70.0) years, and 229 (71.1%) of them were male. There were 250 (77.6%) patients with HTN and 110 (34.2%) with DM.

**Table 1:** Comparison of demographic characteristics between patients with and without CarAC

Characteristics	All (n = 322)	CarAC		*p* value
		With (n = 187)	Without (n = 135)	
Age, years	62.0 (55.0–70.0)	66.0 (58.0–73.0)	57.0 (47.0–64.0)	<*0.001*
Male, n (%)	229 (71.1)	132 (70.6)	97 (71.9)	0.901
BMI, kg/m^2^	24.7 (22.9–26.1)	24.5 (22.6–26.0)	24.9 (23.7–26.4)	*0.032*
HTN, n (%)	250 (77.6)	155 (82.9)	95 (70.4)	*0.010*
DM, n (%)	110 (34.2)	74 (39.6)	36 (26.7)	*0.017*
CAD, n (%)	24 (7.5)	16 (8.6)	8 (5.9)	0.400
TC, mmol/L	4.21 (3.58–5.00)	4.17 (3.40–4.93)	4.28 (3.83–5.12)	*0.029*
TG, mmol/L	1.40 (1.09–1.88)	1.36 (1.03–1.75)	1.54 (1.17–2.02)	*0.016*
HDL, mmol/L	0.98 (0.82–1.15)	0.98 (0.81–1.15)	0.99 (0.84–1.16)	0.363
LDL, mmol/L	2.61 (1.93–3.18)	2.57 (1.79–3.18)	2.68 (2.20–3.19)	0.180
Glucose, mmol/L	5.3 (4.6–6.6)	5.3 (4.7–6.8)	5.2 (4.6–6.2)	0.260
Smoking, n (%)	132 (41.0)	81 (43.3)	51 (37.8)	0.359
Drinking, n (%)	96 (29.8)	58 (31.0)	38 (28.1)	0.622

Statistically signficant values are in italics

Data are presented as number of patients (%) or median (interquartile range)

*CarAC* carotid artery calcification; *BMI* body mass index; *HTN* hypertension; *DM* diabetes mellitus; *CAD* coronary artery disease; *TC* total cholesterol; *TG* triglyceride; *HDL* high-density lipoprotein; *LDL* low-density lipoprotein

Based on Agatston score, 187 (58.1%) patients were grouped as with and 135 (41.9%) without CarAC. CarAC scores presented an extremely left-skewed distribution with a median (interquartile range) of 9.0 (0–111.1). The mean calcium volume (mm^3^) was 11.0 (0–98.0). Compared with patients without CarAC, those with CarAC were older (66.0 vs 57.0 years, *p* < 0.001), and had higher prevalences of HTN (82.9 vs 70.4%, *p* = 0.010) and DM (39.6 vs 26.7%, *p* = 0.017). Patients with CarAC had lower BMI (24.5 vs 24.9, *p* = 0.032), lower TC (4.17 vs 4.28 mmol/L, *p* = 0.029) and lower TG (1.36 vs 1.54 mmol/L, *p* = 0.016) levels (Table 1).

According to the results measured from target regions, there were 36 CpG sites (24 in *CDKN2A* and 12 in *CDKN2B*) identified as methylated sites (detailed information of each site was shown in Additional file 1: Table S2). The distribution of methylation levels of the 36 CpGs were listed in Additional file 1: Table S3. Methylation levels of CpG sites measured within *CDKN2A* were not significantly correlated, while those within *CDKN2B* were significantly correlated (Additional file 1: Table S4 and S5).

The methylation levels of each CpG site and average percent methylation of *CDKN2A/2B* were compared between patients with and without CarAC (Table 2). Higher methylation levels of *CDKN2B* were observed in patients with CarAC (5.7 vs 5.4, *p* = 0.001) compared to those without CarAC. When patients were grouped as with no, mild or severe CarAC, patients with severe CarAC had highest levels of *CDKN2B* (5.4 vs 5.6 vs 5.9, p < 0.001) as shown in Table 3. After adjusting for age, sex, BMI, DM, HTN and smoking, generalized liner model detected a positive correlation between average methylation levels of *CDKN2B* and cube root transformed calcification scores (β = 0.591 ± 0.172, *p* = 0.001, Table 4). And average methylation levels of *CDKN2B* were also associated with (cube root) calcium volumes (β = 0.533 ± 0.160, *p* = 0.001) after the adjustment. When further corrected for multiple comparison, *CDKN2B* methylation levels were still associated with cube root transformed calcification scores (corrected *p* = 0.002) and calcium volumes (corrected *p* = 0.002).

**Table 2:** Differences of methylation levels (%) between patients with and without CarAC

Gene	Position	CarAC		*p* value
		With	Without	
*CDKN2A*	1	4.4 (3.0–6.0)	4.1 (2.7–5.9)	0.480
	2	7.1 (5.3–8.9)	6.7 (5.4–8.5)	0.520
	3	8.2 (6.8–10.7)	8.0 (6.3–9.6)	0.130
	4	5.8 (4.2–8.0)	5.8 (4.3–7.7)	0.896
	5	4.8 (4.0–5.5)	5.1 (4.1–5.5)	0.181
	6	2.7 (2.3–3.4)	2.7 (2.3–3.2)	0.592
	7	2.3 (1.9–2.8)	2.2 (1.7–2.8)	0.062
	8	4.3 (3.8–5.0)	4.4 (3.6–5.2)	0.598
	9	4.4 (2.4–8.2)	4.4 (2.5–7.7)	0.845
	10	2.1 (1.0–3.2)	1.8 (0.9–3.0)	0.377
	11	3.7 (2.5–4.9)	3.5 (2.3–5.0)	0.679
	12	0.9 (0.5–1.3)	0.9 (0.6–1.4)	0.745
	13	1.2 (0.9–1.5)	1.3 (1.0–1.5)	0.325
	14	1.2 (1.0–1.5)	1.2 (0.9–1.4)	0.468
	15	2.1 (1.7–2.3)	2.0 (1.6–2.5)	0.482
	16	1.3 (1.0–1.7)	1.3 (1.0–1.7)	0.800
	17	3.3 (2.7–4.2)	3.1 (2.6–3.5)	*0.001*
	18	2.2 (1.7–2.6)	2.2 (1.8–2.6)	0.937
	19	2.5 (2.0–3.0)	2.5 (2.0–2.9)	0.806
	20	2.7 (2.1–3.3)	2.7 (2.3–3.2)	0.615
	21	15.6 (13.6–17.8)	15.3 (13.9–16.8)	0.511
	22	2.5 (2.0–3.1)	2.6 (2.1–3.2)	0.425
	23	4.3 (3.5–5.1)	4.3 (3.5–5.0)	0.983
	24	1.7 (1.2–2.5)	1.8 (1.3–2.5)	0.449
	Average	4.0 (3.6–4.3)	3.9 (3.6–4.2)	0.277
*CDKN2B*	1	5.5 (4.5–6.4)	5.2 (4.2–6.1)	0.046
	2	4.4 (3.4–5.3)	4.3 (3.3–5.2)	0.395
	3	3.8 (3.1–4.9)	4.0 (3.1–4.6)	0.890
	4	4.2 (3.4–5.1)	4.1 (3.3–4.8)	0.233
	5	7.6 (6.6–8.9)	7.2 (6.3–8.1)	0.002
	6	6.9 (5.7–8.2)	6.5 (5.3–7.4)	0.009
	7	8.3 (7.1–9.6)	7.5 (6.7–8.2)	<*0.001*
	8	3.5 (2.9–4.2)	3.3 (2.9–3.7)	0.039
	9	4.0 (3.3–4.5)	3.6 (3.0–4.1)	<*0.001*
	10	6.1 (5.2–7.1)	5.6 (4.6–6.3)	<*0.001*
	11	7.5 (6.3–8.7)	6.8 (6.0–7.8)	0.007
	12	5.7 (4.9–6.7)	5.4 (4.6–6.0)	0.009
	Average	5.7 (5.0–6.4)	5.4 (4.7–5.9)	*0.001*

Statistically signficant values are in italics

For each CpG site, *p* < 0.05/36 after Bonferroni correction, and *p* **<** 0.025 for average levels

**Table 3:** Methylation levels of *CDKN2A/2B* according to severity of CarAC

Gene	Without (n = 135)	Mild (n = 103)	Sever (n = 84)	*p* value
*CDKN2A*	3.9 (3.6–4.2)	4.0 (3.6–4.4)	4.0 (3.6–4.2)	0.189
*CDKN2B*	5.4 (4.7–5.9)	5.6 (4.8–6.2)	5.9 (5.2–6.6)	<*0.001*

Statistically signficant values are in italics

**Table 4:** Association between methylation levels of *CDKN2A/2B* and cube root transformed calcification scores/calcium volumes

	Agatston score			Calcium volume		
	β	SE	*p* value	β	SE	*p* value
*Model 1*						
*CDKN2A*	0.013	0.325	0.968	0.032	0.302	0.915
Age	0.108	0.016	<*0.001*	0.101	0.015	<*0.001*
Sex	0.566	0.461	0.219	0.556	0.429	0.194
BMI	−0.136	0.064	*0.034*	−0.130	0.059	*0.029*
HTN	1.173	0.429	*0.006*	1.084	0.399	*0.007*
DM	0.844	0.377	*0.025*	0.792	0.351	*0.024*
Smoking	0.654	0.404	0.105	0.599	0.375	0.111
*Model 2*						
*CDKN2B*	0.591	0.172	*0.001*	0.533	0.160	*0.001*
Age	0.078	0.018	<*0.001*	0.074	0.017	<*0.001*
Sex	0.433	0.451	0.338	0.437	0.420	0.299
BMI	−0.127	0.062	*0.042*	−0.122	0.058	*0.036*
HTN	1.348	0.420	*0.001*	1.240	0.392	*0.002*
DM	0.750	0.367	*0.041*	0.704	0.342	*0.039*
Smoking	0.746	0.392	0.057	0.678	0.365	0.063

Statistically signficant values are in italics

Generalized liner model was adjusted for age, sex, BMI, HTN, DM and smoking

## Discussion

In this study, we observed a positive correlation between *CDKN2B* methylation and CarAC, which was quantified by Agatston score and calcium volume. These results verified our hypothesis that DNA methylation in *CDKN2B* may increase the susceptibility of artery calcification.

The relationship between Chr9p21 variants and artery calcification has been established previously [9, 23, 24]. Chr9p21 variants may up-regulate the expression of *ANRIL*, which was negatively correlated with the expression of *CDKN2B* [25]. *ANRIL* can recruit and bind epigenetic modifiers such as polycomb repressor complex to promoter regions of adjacent genes [12, 15, 26]. These epigenetic regulations may eventually influence DNA methylation of *CDKN2B*. Methylation occurred in CpG islands around promoter regions generally inhibits gene expression [27]. *CDKN2B*, known as a tumor suppressor, participates in cell cycle regulation via retinoblastoma (Rb) pathway [28]. The protein p15^INK4b^, encoded by *CDKN2B*, can specifically bind to CDKN4 and CDKN6, resulting in G1 phase arrest and blockage of cell proliferation [8]. The viewpoint that *CDKN2B* methylation may lead to unlimited cell proliferation has been verified in a spectrum of cancers [29, 30].

Chronic vascular inflammation arising from atherosclerosis contributes to calcification [6]. Repression of CDKN2B may result in losing control of Rb proteins, which may subsequently enhance the proliferation of macrophage [12]. In the condition of imbalance between promotion and inhibition of calcification, a proportion of VSMCs tend to differentiate into an osteoblastic and proliferative phenotype [31–33]. These processes play a role in the progression of arterial calcification. Therefore, methylation at *CDKN2B* may be a substantial contributor to artery calcification. And the possible association of *CDKN2B* methylation and atherosclerosis can be further extrapolated to patients with CAD or other cardiovascular diseases.

Our study has several strengths. To the best of our knowledge, this study was the first to report the association between *CDKN2B* methylation status and CarAC. CarAC was quantified by both Agatston method and calcium volume. Considering its less invasiveness and simplicity, methylation tests may be used in clinical settings for predicting the artery calcification. There are potential treatment implications. CpG island hypermethylation has been targeted in cancer treatment, with pharmacological agents modifying the epigenetic mechanisms been studied intensively [30]. Similarly, agents which can specifically regulate *CDKN2B* methylation may be used for preventing artery calcification in future.

There are several limitations in our study. Firstly, the nature of the cross-sectional study limited us to reach a causal relationship. Secondly, the *CDKN2A/2B* expression was not evaluated in this study due to lack of fresh leukocytes. Further functional studies are warranted to clarify the underlying mechanisms that correlate *CDKN2B* methylation with artery calcification. Third, given the varied predisposition of DNA methylation in different tissues, methylation measured from leukocytes may not represent that of arterial wall. But considering that monocyte-derived macrophages, lymphocytes and platelets from peripheral blood are involved in atherogenesis [34], and harvesting vascular tissue from human body is largely impractical, the research strategy used in this study is logical and rational. Fourth, the present study was conducted in patients with ischemic stroke, which may generate selection bias. Not all potential confounders can be collected and analyzed due to the limited sample size and study resource. Moreover, patients with history of carotid artery stenting were excluded for accurate calcification assessment, which may lead to selection bias.

## Conclusions

In summary, *CDKN2B* methylation is associated with CarAC independent of major cardiovascular risk factors. Our findings may enrich the body of knowledge on epigenetic pathology and provide some new implications for prevention and treatment of atherosclerotic diseases.

## Additional file

**Additional file 1: Table S1.** Primer sequences for *CDKN2A/2B* genes (start and end site were named as its relative distance to transcriptional start site). **Table S2.** Methylated CpG sites identified in this study. **Table S3.** Distribution of methylation levels (%) of 36 CpG sites in *CDKN2A/2B* genes. **Table S4.** Spearman pairwise correlations for CpG sites of *CDKN2A.* **Table S5.** Spearman pairwise correlations for CpG sites of *CDKN2B.*

## Abbreviations

ANRIL: antisense noncoding RNA in the INK4 locus
BMI: body mass index
BSAS: BiSulfite Amplicon Sequencing
CarAC: carotid artery calcification
CAD: coronary artery disease
CDKN2A/2B: cyclin-dependent kinase inhibitor 2A/2B
Chr9p21: chromosome 9p21
CTA: computed tomography angiography
DM: diabetes mellitus
HDL: high-density lipoprotein
HTN: hypertension
HU: Hounsfield units
LDL: low-density lipoprotein
Rb: retinoblastoma
TC: total cholesterol
TG: triglyceride
TSS: transcriptional start site
VSMC: vascular smooth muscle cell
