# Supplementary material for: CDKN2B methylation is associated with carotid artery calcification in ischemic stroke patients
Source: J Transl Med. 2016 Dec 1;14:333. doi: 10.1186/s12967-016-1093-4 (PMC5134267; doi:10.1186/s12967-016-1093-4)
Supplement: Supplementary file 1 — Additional file 1: Table S1. Primer sequences for CDKN2A/2B genes (start and end site were named as its relative distance to transcriptional start site). Table S2. Methylated CpG sites identified in this study. Table S3. Distribution of methylation levels (%) of 36 CpG sites in CDKN2A/2B genes. Table S4. Spearman pairwise correlations for CpG sites of CDKN2A. Table S5. Spearman pairwise correlations for CpG sites of CDKN2B. [file 12967_2016_1093_MOESM1_ESM.docx]

**Supplementary Material**

***CDKN2B* Methylation Is Associated With Carotid Artery Calcification in Ischemic Stroke Patients**

Shuyu Zhou, Yumeng Zhang, Li Wang, Zhizhong Zhang, Biyang Cai, Keting Liu, Hao Zhang, Minhui Dai, Lingli Sun, Xiaomeng Xu, Huan Cai, Xinfeng Liu, Guangming Lu, Gelin Xu

**Table S1.** Primer sequences for *CDKN2A/2B* genes (start and end site were named as its relative distance to transcriptional start site)

| Gene | PCR  size(bp) | Start site | End  site | Primer |  |
| --- | --- | --- | --- | --- | --- |
| *CDKN2A* | 282 | -1477 | -1197 | forward | GGGATATGGAGGGGGAGAT |
|  |  |  |  | reverse | CTTCTTCCTCTTTCCTCTTCCC |
|  | 211 | -1047 | -838 | forward | GGGAAGAGGAAAGAGGAAGAAG |
|  |  |  |  | reverse | ATTAAACTAAACCRCTACACRCCTCTAAC |
|  | 286 | -859 | -574 | forward | AATAAAATAAGGGGAATAGGGGAG |
|  |  |  |  | reverse | CCATCTTCCCACCCTCAA |
|  | 188 | -399 | -212 | forward | GTAGTTAAGGGGGTAGGAGTGG |
|  |  |  |  | reverse | ACTACTACCCTAAACRCTAACTCCTCAA |
|  | 266 | +70 | +335 | forward | TTGAGGAGTTAGYGTTTAGGGTAGTAGT |
|  |  |  |  | reverse | TCAATAATACTACRAAAACCACATATCTAAATC |
|  | 224 | +308 | +531 | forward | GTYGGTTGGTTTTTTATTTTGTTAGAG |
|  |  |  |  | reverse | AACCTAAACTCAACTTCATTACCCTC |
| *CDKN2B* | 255 | -7 | +248 | forward | GAGGGTAATGAAGTTGAGTTTAGGTT |
|  |  |  |  | reverse | CTATCRCACCTTCTCCACTAATCC |
|  | 234 | +223 | +455 | forward | GGGGATTAGTGGAGAAGGTG |
|  |  |  |  | reverse | TAAAATACACACCTCCRACCAAC |
|  | 221 | +430 | +650 | forward | TGTTTTTTAAGTTTTTATAGGGTGAGG |
|  |  |  |  | reverse | CCAACCTAACCAAAATAATAAAAACC |

**Table S2.** Methylated CpG sites identified in this study.

| Gene | Position | Genomic location* | Relative to TSS, bp |
| --- | --- | --- | --- |
| *CDKN2A* | 1 | Chr9: 21995909 | -1419 |
|  | 2 | Chr9: 21995896 | -1406 |
|  | 3 | Chr9: 21995867 | -1377 |
|  | 4 | Chr9: 21995713 | -1223 |
|  | 5 | Chr9: 21995470 | -980 |
|  | 6 | Chr9: 21995457 | -967 |
|  | 7 | Chr9: 21995455 | -965 |
|  | 8 | Chr9: 21995354 | -864 |
|  | 9 | Chr9: 21995314 | -824 |
|  | 10 | Chr9: 21995312 | -822 |
|  | 11 | Chr9: 21995305 | -815 |
|  | 12 | Chr9: 21995108 | -618 |
|  | 13 | Chr9: 21994859 | -369 |
|  | 14 | Chr9: 21994782 | -292 |
|  | 15 | Chr9: 21994734 | -244 |
|  | 16 | Chr9: 21994727 | -237 |
|  | 17 | Chr9: 21994286 | +205 |
|  | 18 | Chr9: 21994215 | +276 |
|  | 19 | Chr9: 21994211 | +280 |
|  | 20 | Chr9: 21994208 | +283 |
|  | 21 | Chr9: 21994155 | +336 |
|  | 22 | Chr9: 21994109 | +382 |
|  | 23 | Chr9: 21994076 | +415 |
|  | 24 | Chr9: 21993993 | +498 |
| *CDKN2B* | 1 | Chr9: 22009259 | +54 |
|  | 2 | Chr9: 22009179 | +134 |
|  | 3 | Chr9: 22009165 | +148 |
|  | 4 | Chr9: 22009134 | +179 |
|  | 5 | Chr9: 22009000 | +313 |
|  | 6 | Chr9: 22008981 | +332 |
|  | 7 | Chr9: 22008956 | +357 |
|  | 8 | Chr9: 22008890 | +423 |
|  | 9 | Chr9: 22008845 | +468 |
|  | 10 | Chr9: 22008830 | +483 |
|  | 11 | Chr9: 22008815 | +498 |
|  | 12 | Chr9: 22008804 | +509 |

*The chromosomal location of each CpG site according to assembly GRCh37/hg19.

**Table S3.** Distribution of methylation levels (%) of 36 CpG sites in *CDKN2A/2B* genes.

| Gene | Position | Min | Q1 | Median | Q3 | Max |
| --- | --- | --- | --- | --- | --- | --- |
| *CDKN2A* | 1 | 0.0 | 2.8 | 4.3 | 5.9 | 17.2 |
|  | 2 | 0.0 | 5.3 | 7.0 | 8.8 | 19.7 |
|  | 3 | 0.0 | 6.5 | 8.1 | 10.2 | 21.4 |
|  | 4 | 0.0 | 4.3 | 5.8 | 7.9 | 18.6 |
|  | 5 | 0.0 | 4.1 | 4.9 | 5.5 | 9.7 |
|  | 6 | 0.0 | 2.3 | 2.7 | 3.3 | 9.2 |
|  | 7 | 0.0 | 1.8 | 2.3 | 2.8 | 7.7 |
|  | 8 | 1.8 | 3.7 | 4.4 | 5.0 | 10.4 |
|  | 9 | 0.0 | 2.4 | 4.4 | 7.9 | 23.5 |
|  | 10 | 0.0 | 1.0 | 2.0 | 3.1 | 8.8 |
|  | 11 | 0.0 | 2.4 | 3.6 | 4.9 | 13.3 |
|  | 12 | 0.0 | 0.6 | 0.9 | 1.3 | 2.9 |
|  | 13 | 0.0 | 1.0 | 1.2 | 1.5 | 2.6 |
|  | 14 | 0.0 | 1.0 | 1.2 | 1.4 | 2.9 |
|  | 15 | 0.0 | 1.6 | 2.1 | 2.4 | 6.9 |
|  | 16 | 0.0 | 1.0 | 1.3 | 1.7 | 3.4 |
|  | 17 | 0.0 | 2.6 | 3.2 | 3.8 | 25.3 |
|  | 18 | 0.5 | 1.7 | 2.2 | 2.6 | 7.5 |
|  | 19 | 0.0 | 2.0 | 2.5 | 3.0 | 7.5 |
|  | 20 | 0.4 | 2.2 | 2.7 | 3.2 | 8.9 |
|  | 21 | 4.9 | 13.7 | 15.5 | 17.2 | 34.2 |
|  | 22 | 0.7 | 2.1 | 2.6 | 3.2 | 8.6 |
|  | 23 | 0.0 | 3.5 | 4.3 | 5.1 | 11.0 |
|  | 24 | 0.0 | 1.3 | 1.7 | 2.5 | 6.2 |
|  | Average | 2.4 | 3.6 | 3.9 | 4.3 | 6.2 |
| *CDKN2B* | 1 | 1.8 | 4.4 | 5.4 | 6.2 | 12.2 |
|  | 2 | 0.0 | 3.4 | 4.4 | 5.2 | 10.9 |
|  | 3 | 0.0 | 3.1 | 3.9 | 4.8 | 9.3 |
|  | 4 | 0.0 | 3.3 | 4.1 | 5.0 | 18.8 |
|  | 5 | 4.4 | 6.4 | 7.4 | 8.6 | 16.4 |
|  | 6 | 3.4 | 5.5 | 6.7 | 7.8 | 13.1 |
|  | 7 | 4.1 | 6.8 | 7.9 | 9.1 | 18.5 |
|  | 8 | 0.9 | 2.9 | 3.4 | 4.0 | 12.1 |
|  | 9 | 0.0 | 3.2 | 3.7 | 4.4 | 17.1 |
|  | 10 | 0.0 | 5.0 | 5.9 | 6.7 | 18.0 |
|  | 11 | 3.7 | 6.2 | 7.1 | 8.5 | 27.9 |
|  | 12 | 2.4 | 4.8 | 5.5 | 6.5 | 23.9 |
|  | Average | 3.5 | 4.8 | 5.5 | 6.2 | 11.1 |

Q1: 1st quartile (25th percentile), Q3: 3rd quartile (75th percentile).

**Table S4.** Spearman pairwise correlations for CpG sites of *CDKN2A*.

| Position | 1 | 2 | 3 | 4 | 5 | 6 | 7 | 8 | 9 | 10 | 11 | 12 | 13 | 14 | 15 | 16 | 17 | 18 | 19 | 20 | 21 | 22 | 23 | 24 |
| --- | --- | --- | --- | --- | --- | --- | --- | --- | --- | --- | --- | --- | --- | --- | --- | --- | --- | --- | --- | --- | --- | --- | --- | --- |
| 1 | 1.0 | 0.5* | 0.4* | 0.4* | 0.0 | 0.0 | 0.0 | 0.0 | 0.0 | 0.0 | 0.0 | -0.1* | 0.1 | -0.1 | 0.0 | -0.1 | -0.1 | -0.1 | 0.0 | -0.1* | 0.0 | 0.1 | 0.1 | 0.2* |
| 2 |  | 1.0 | 0.5* | 0.4* | 0.0 | 0.0 | 0.0 | 0.1* | 0.0 | 0.0 | 0.0 | 0.0 | -0.1 | -0.1 | 0.1 | -0.1* | 0.0 | 0.0 | 0.0 | 0.0 | 0.1 | 0.1 | 0.1* | 0.3* |
| 3 |  |  | 1.0 | 0.4* | 0.1 | 0.0 | 0.0 | 0.1* | 0.0 | 0.0 | 0.0 | 0.0 | 0.0 | 0.0 | 0.1 | -0.1 | 0.1 | 0.1 | 0.1 | 0.1 | 0.1 | 0.1* | 0.2* | 0.3* |
| 4 |  |  |  | 1.0 | 0.1 | 0.1 | 0.0 | 0.1 | 0.1 | 0.0 | 0.0 | -0.1 | 0.1 | 0.0 | 0.1 | 0.0 | 0.1 | 0.0 | 0.0 | 0.0 | 0.0 | 0.1 | 0.1 | 0.2* |
| 5 |  |  |  |  | 1.0 | 0.2* | 0.3* | 0.2* | 0.0 | 0.0 | 0.0 | 0.1 | 0.2* | 0.1 | 0.1 | 0.2* | 0.0 | 0.1 | 0.1 | 0.0 | 0.0 | 0.0 | 0.0 | 0.1* |
| 6 |  |  |  |  |  | 1.0 | 0.3* | 0.3* | -0.1 | 0.1 | 0.1 | 0.1* | 0.1 | 0.2* | 0.2* | 0.2* | 0.1 | 0.1* | 0.1 | 0.0 | 0.0 | 0.1* | 0.1 | 0.2* |
| 7 |  |  |  |  |  |  | 1.0 | 0.2* | 0.0 | 0.1 | 0.0 | 0.1 | 0.0 | 0.1 | 0.2* | 0.2* | 0.0 | 0.1 | 0.1 | 0.0 | 0.0 | 0.0 | 0.0 | 0.1 |
| 8 |  |  |  |  |  |  |  | 1.0 | 0.1 | 0.1 | 0.1 | 0.2* | 0.2* | 0.1 | 0.3* | 0.2* | 0.1* | 0.2* | 0.1 | 0.1* | 0.0 | 0.1* | 0.1 | 0.1 |
| 9 |  |  |  |  |  |  |  |  | 1.0 | 0.0 | -0.1 | 0.0 | 0.0 | 0.0 | 0.0 | 0.0 | 0.0 | -0.1 | -0.1 | -0.1* | -0.1* | 0.0 | 0.0 | 0.0 |
| 10 |  |  |  |  |  |  |  |  |  | 1.0 | 0.1 | 0.0 | -0.1 | 0.1 | 0.0 | 0.0 | 0.0 | 0.0 | 0.0 | 0.1 | 0.0 | -0.1 | 0.0 | 0.1 |
| 11 |  |  |  |  |  |  |  |  |  |  | 1.0 | 0.1 | 0.0 | 0.0 | 0.0 | 0.1 | 0.1 | 0.1 | 0.1 | 0.0 | 0.1 | 0.2* | 0.0 | 0.0 |
| 12 |  |  |  |  |  |  |  |  |  |  |  | 1.0 | 0.0 | 0.0 | 0.1* | 0.1 | 0.1 | 0.1 | 0.1 | 0.1 | 0.0 | 0.1 | 0.0 | -0.1 |
| 13 |  |  |  |  |  |  |  |  |  |  |  |  | 1.0 | 0.1 | 0.0 | 0.1* | 0.0 | 0.0 | 0.0 | 0.0 | 0.0 | 0.0 | 0.0 | 0.0 |
| 14 |  |  |  |  |  |  |  |  |  |  |  |  |  | 1.0 | 0.1* | 0.1 | 0.1 | 0.1 | 0.1 | 0.1* | 0.0 | 0.1* | 0.1* | 0.0 |
| 15 |  |  |  |  |  |  |  |  |  |  |  |  |  |  | 1.0 | 0.1 | 0.0 | 0.1 | 0.0 | 0.1 | 0.0 | 0.1 | 0.1 | 0.2* |
| 16 |  |  |  |  |  |  |  |  |  |  |  |  |  |  |  | 1.0 | 0.1 | 0.0 | 0.2* | 0.1 | 0.1 | 0.0 | 0.0 | 0.1 |
| 17 |  |  |  |  |  |  |  |  |  |  |  |  |  |  |  |  | 1.0 | 0.1* | 0.2* | 0.3* | 0.2* | 0.1* | 0.2* | 0.0 |
| 18 |  |  |  |  |  |  |  |  |  |  |  |  |  |  |  |  |  | 1.0 | 0.2* | 0.3* | 0.2* | 0.1 | 0.0 | 0.1 |
| 19 |  |  |  |  |  |  |  |  |  |  |  |  |  |  |  |  |  |  | 1.0 | 0.3* | 0.1* | 0.0 | 0.1* | 0.1 |
| 20 |  |  |  |  |  |  |  |  |  |  |  |  |  |  |  |  |  |  |  | 1.0 | 0.2* | 0.1 | 0.1* | 0.0 |
| 21 |  |  |  |  |  |  |  |  |  |  |  |  |  |  |  |  |  |  |  |  | 1.0 | 0.1* | 0.1 | 0.1 |
| 22 |  |  |  |  |  |  |  |  |  |  |  |  |  |  |  |  |  |  |  |  |  | 1.0 | 0.2* | 0.3* |
| 23 |  |  |  |  |  |  |  |  |  |  |  |  |  |  |  |  |  |  |  |  |  |  | 1.0 | 0.2* |
| 24 |  |  |  |  |  |  |  |  |  |  |  |  |  |  |  |  |  |  |  |  |  |  |  | 1.0 |

**p* < 0.05

**Table S5.** Spearman pairwise correlations for CpG sites of *CDKN2B*.

| Position | 1 | 2 | 3 | 4 | 5 | 6 | 7 | 8 | 9 | 10 | 11 | 12 |
| --- | --- | --- | --- | --- | --- | --- | --- | --- | --- | --- | --- | --- |
| 1 | 1.0 | 0.5 | 0.5 | 0.4 | 0.6 | 0.6 | 0.6 | 0.3 | 0.3 | 0.4 | 0.5 | 0.4 |
| 2 |  | 1.0 | 0.5 | 0.5 | 0.5 | 0.5 | 0.5 | 0.3 | 0.3 | 0.4 | 0.3 | 0.3 |
| 3 |  |  | 1.0 | 0.4 | 0.5 | 0.5 | 0.5 | 0.3 | 0.3 | 0.3 | 0.3 | 0.3 |
| 4 |  |  |  | 1.0 | 0.5 | 0.5 | 0.5 | 0.3 | 0.3 | 0.3 | 0.4 | 0.4 |
| 5 |  |  |  |  | 1.0 | 0.8 | 0.7 | 0.4 | 0.4 | 0.6 | 0.5 | 0.5 |
| 6 |  |  |  |  |  | 1.0 | 0.8 | 0.4 | 0.4 | 0.6 | 0.5 | 0.6 |
| 7 |  |  |  |  |  |  | 1.0 | 0.3 | 0.4 | 0.5 | 0.5 | 0.5 |
| 8 |  |  |  |  |  |  |  | 1.0 | 0.3 | 0.3 | 0.3 | 0.3 |
| 9 |  |  |  |  |  |  |  |  | 1.0 | 0.6 | 0.6 | 0.5 |
| 10 |  |  |  |  |  |  |  |  |  | 1.0 | 0.8 | 0.7 |
| 11 |  |  |  |  |  |  |  |  |  |  | 1.0 | 0.7 |
| 12 |  |  |  |  |  |  |  |  |  |  |  | 1.0 |

All *p* < 0.001
